# First molecular identification of mosquito vectors of *Dirofilaria immitis* in continental Portugal

**DOI:** 10.1186/s13071-015-0760-2

**Published:** 2015-03-03

**Authors:** Cátia Alexandra Costa Ferreira, Verónica de Pinho Mixão, Maria Teresa Lourenço Marques Novo, Maria Manuela Palmeiro Calado, Luzia Augusta Pires Gonçalves, Silvana Maria Duarte Belo, António Paulo Gouveia de Almeida

**Affiliations:** Medical Parasitology Unit, Medical Parasitology & Microbiology Unit-UPMM, Instituto de Higiene e Medicina Tropical, Universidade Nova de Lisboa, Rua da Junqueira 100, 1349-008 Lisboa, Portugal; Zoonosis Research Unit, Faculty of Health Sciences, University of Pretoria, Pretoria, South Africa; International Public Health and Biostatistics Unit, Instituto de Higiene e Medicina Tropical, Universidade Nova de Lisboa, Rua da Junqueira 100, 1349-008 Lisboa, Portugal; Centro de Estatística e Aplicações da Universidade de Lisboa, Faculdade de Ciências da Universidade de Lisboa, Bloco C6 - Piso 4, Campo Grande, 1749-016 Lisboa Portugal

**Keywords:** *Dirofilaria*, Mosquitoes, Vectors, Portugal, DDU, *Cx. theileri*, *Cx. pipiens*, *An. maculippenis* s.l, *Ae. caspius*, *Ae. detritus* s.l

## Abstract

**Background:**

Canine dirofilariasis due to *Dirofilaria immitis* is known to be endemic in continental Portugal. However, information about the transmitting mosquito species is still scarce, with only *Culex theileri* identified to date, albeit with L1-2, through dissection. This study was carried out to investigate the potential vectors of *Dirofilaria* spp. in continental Portugal.

**Methods:**

Mosquitoes were collected in three distinct seasons (Summer, Autumn and Spring), 2011–2013, in three districts. CDC traps and indoor resting collections were carried out in the vicinity of kennels. Mosquitoes were kept under controlled conditions for 7 days to allow the development of larval stages of *Dirofilaria* spp*.*. DNA extraction was performed separately for both head+thorax and abdomen in order to differentiate infective and infected specimens, respectively, in pools, grouped according to the species and collection site (1–40 specimen parts/pool), and examined by PCR using pan-filarial specific primers. Mosquito densities were compared using non-parametric tests. *Dirofilaria* development units (DDU) were estimated.

**Results:**

In total, 9156 female mosquitoes, from 11 different species, were captured. Mosquito densities varied among the 3 districts, according to capture method, and were generally higher in the second year of collections. From 5866 specimens screened by PCR, 23 head+thorax and 41 abdomens pools, corresponding to 54 mosquitoes were found positive for *D. immitis* DNA. These belonged to 5 species: *Culex (Cux) theileri* (estimated rate of infection (ERI)=0.71%), *Cx. (Cux) pipiens* f. *pipiens* and f. *molestus* (ERI=0.5%), *Anopheles (Ano) maculipennis* s.l. (ERI=3.12%), including *An. (Ano) atroparvus*, *Aedes (Och) caspius* (ERI=3.73%) and *Ae. (Och) detritus* s.l. (ERI=4.39%). All but *Cx. pipiens*, had at least one infective specimen. No *D. repens* infected specimens were found. Infection rates were: 3.21% in Coimbra, 1.22% in Setúbal and 0.54% in Santarém. DDU were at least 117/year in the study period.

**Conclusions:**

*Culex theileri*, *Cx. pipiens*, *An. maculipennis* s.l. *An. atroparvus, Ae.caspius* and *Ae. detritus* s.l. were identified as potential vectors of *D. immitis* in three districts of Portugal, from Spring to Autumn, in 5 of the 6 collection dates in 2011–2013. Implications for transmission, in the context of climate changes, and need for prophylactic measures, are discussed.

## Background

Dirofilariasis is a mosquito-borne cosmopolitan metazoonotic disease caused by different species of the nematode genus *Dirofilaria* (Spirurida: Onchocercidae) [1], namely *Dirofilaria immitis* (Leidy, 1856), canine or dog heartworm, and *Dirofilaria repens* Railliet& Henry, 1911.

Although the natural hosts of *Dirofilaria* spp. are dogs and wild members of the genus *Canis*, canine dirofilariasis (CD) infections may occur in a variety of species, including cats, other wild mammals and humans [2,3]. Previously human dirofilariasis (HD) was considered a rare disease, but a recent increase in the number of CD and HD cases, particularly after 2000, has resulted in it being classified as an emerging zoonosis [4,5]. Recent accounts of autochthonous cases of CD have stemmed from Slovakia [6], Hungary [7], Poland [8], and of HD from Hungary [9], Poland [10], Ukraine [11], and seroreactivity prevalences ranging from 5%-27% amongst humans, have been recorded in Serbia [12].

*Dirofilaria* spp. are transmitted by several mosquito species belonging to a wide range of genera in different parts of the world, such as *Culex, Aedes* and *Anopheles* [5]. Vectors ingest microfilariae, while feeding on an infected host, which then cross the midgut wall and migrate to the Malpighian tubules (MT) where they develop from first to third stage larvae. Later, the L3 (infective larvae) migrate to the proboscis through which they slide while the mosquito is feeding on another host, becoming sexually mature within six months in the main pulmonary arteries and right ventricle [1]. Transmission of dirofilariasis is dependent upon the presence of sufficient numbers of infected and microfilaremic dogs, susceptible mosquitoes, and a suitable climate to allow extrinsic incubation of the parasite in the mosquito vector [13,14]. Environmental factors, namely climatic and ecological, may affect the life cycle parameters of both the mosquito vector and filarial parasites.

Various studies in European countries and neighbouring areas have reported several species of mosquitoes as natural vectors of *D. immitis* such as *Culex (Culex) pipiens* in Spain [15], Italy [16,17], and [18], *Aedes (Ochlerotatus) vexans* in Turkey [18], *Cx. (Cux) theileri* on Madeira and Canary Islands [19,20], *Cx. theileri* and *Anopheles (Anopheles) maculipennis* s.l. in Iran [21] and *Aedes (Stegomyia) albopictus*, *An. maculipennis* s.l. and *Coquillettidia (Coquillettidia) richiardii* in Italy [16,22].

Canine dirofilariasis due to *D. immitis* is known to be endemic in continental Portugal. In 2009–2010 the overall sero-prevalence in Northern and Central Portugal was 2.1% for CD [23]. A recent survey, 2011–2013, in three districts of Centre-South, has revealed an overall parasitological prevalence rate of 15.1%, the highest in Setúbal (24.8%), followed by Coimbra (13.8%) and Santarém (13.2%) [24].

Despite these high prevalences, information about the transmitting mosquito species was still scarce in continental Portugal, with *Cx. theileri* as the only likely vector of *Dirofilaria* spp. [25]. In addition, high densities of mosquito populations, namely *Cx. theileri*, *Cx. pipiens* s.l. *An. maculipennis* s.l*.* and *Ae.caspius*, were recorded in the above mentioned areas [26]. Hence, the purpose of this study was to identify potential vectors of *Dirofilaria* spp. by using a polymerase chain reaction (PCR) with species specific primers on mosquito populations from those three districts of continental Portugal, Coimbra, Santarém and Setúbal, collected in the vicinity of kennels being surveyed for CD, in a multidisciplinary project, for a period of two consecutive years.

## Methods

### Sampling area

**Figure 1:**
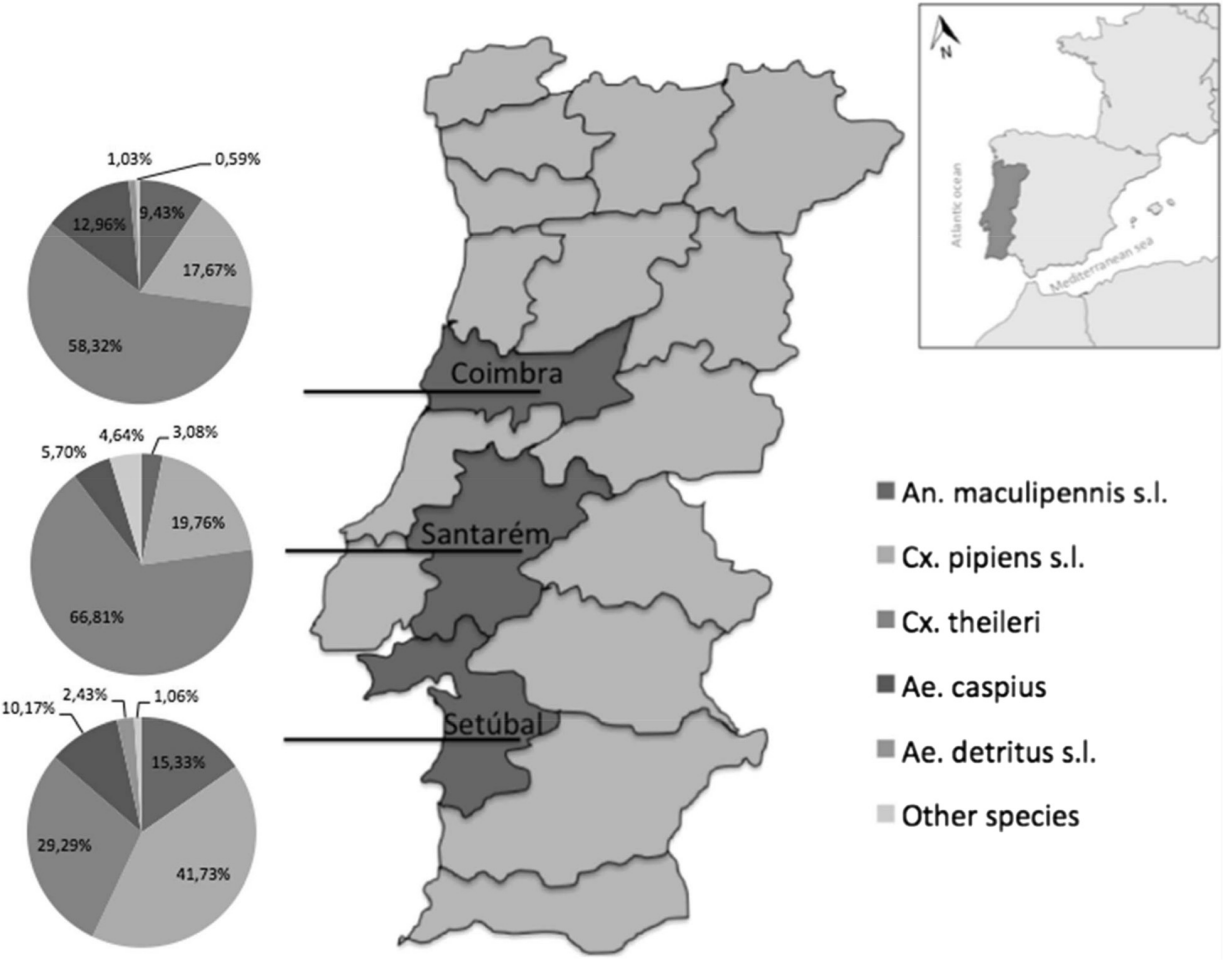
**Relative abundance by district of total adult female mosquitoes collected in mainland Portugal over 2011–2013.**

The research was concentrated on three districts of Portugal: Coimbra (Centre), Santarém, and Setúbal (Centre-South), located at the basins of rivers Mondego, Tejo and Sado, respectively (Figure 1). These districts present different prevalences of dog infections, ecological and overall soil use, although they have in common the presence of the main rice culture areas in the country. The number of localities surveyed in each district was, Coimbra- four, Santarém- five, and Setúbal- four.

Daily temperature data of 2011, 2012 and 2013 from stations operated close to the collections sites, were obtained from “Instituto Português do Mar e da Atmosfera” [27]. Average minimum and maximum monthly temperatures and rainfall values, for the study period are depicted in Table 1.

**Table 1:** **Average minimum and maximum, monthly temperatures and rainfall values, standard deviations, and respective months** [27]

**2011-2013**	**Coimbra**	**Santarém**	**Setúbal**
Minimum average temperature	9.3±2.6°C (Feb)	11.7±2.0°C (Jan)	11±2.1°C (Feb)
Maximum average temperature	20.4±1.8°C (Aug)	24.8±2.4°C (Aug)	23.3±1.6°C (Aug)
Maximum average rainfall	1.83±5.91 mm (Oct)	1.65±4.79 mm (Mar)	1±3.52 mm (Oct)
Minimum average rainfall	0.02±0.12 mm (Jul)	0±0 mm (Jul/Aug)	0±0 mm (Jul)

### Mosquito collection and identification

Mosquitoes were collected by CDC light traps baited with dry ice, between 5.00 p.m. and 7.00 a.m., for active adult mosquitoes and with mechanical aspirators in the early morning targeting indoor resting mosquitoes (IR). Collections were carried out in kennels (whose identities are confidential) or their vicinity, but also in suburban or rural areas in those districts. Collections were carried out from 2011 to 2013, in July, October-November and April-May corresponding to Summer, Autumn and Spring seasons. Mosquitoes were kept in the insectary under controlled conditions of temperature and humidity (25±2°C, 70±5% RH), a photoperiod of 12 h:12 h (light:dark) and fed 10% sucrose solution, for 7 days to allow bloodmeal digestion and eventual parasite development to the infective L3 stage [1], as done in other studies [22]. After this period, those specimens still alive were frozen until species identification was carried out according to the keys of Ribeiro & Ramos [28]. Mosquitoes that were dead at time of trap collection, or that died during the following seven day period, were also frozen, identified and screened for filarial infection.

### Set of biological material for PCR analysis

Mosquito females were dissected into head+thorax and abdomen to discriminate between *Dirofilaria* spp. infective/infected status, respectively [29]. Specimens belonging to the same collection, species, in identical gonotrophic stage, that had been the same number of days in the insectary, were joined in pools of these body parts, ranging from 1 to 40 specimen parts. Specimens of *Cx. pipiens* s.l. were individually analyzed, due to the sympatric existence of the two biological forms, *pipiens* and *molestus* of the *sensu strictu* species, in Portugal [30].

### DNA isolation

Genomic DNA was extracted from samples using the CTAB (Cetyltrimethylammonium bromide) method, adapted from Stothard et al. [31] by grinding the mosquito samples in a buffer (100 mMTris, 1.4 M NaCl, 20 mM EDTA, 2% Hexadecyltrimethylammonium bromide (CTAB), 0.2% mercaptoethanol) and incubating with proteinase K (Bioline) at 55°C for 90 min with agitation. Phenol/chloroform/isoamyl alcohol was used for further DNA purification. DNA was ethanol precipitated and pellet was suspended in TE buffer (pH 7.0).

### DNA amplification

DNA from head+thorax and abdomen samples were analyzed by panfilarial primers DIDR-F1 (5’-AGT GCG AAT TGC AGA CGC ATT GAG-3’) and DIDR-R1 (5’-AGC GGG TAA TCA CGA CTG AGT TGA-3’), described by Rishniw et al. [32]. PCR was performed in 25 μl reaction mixture containing a final concentration of 1 X GreenGoTaq® Flexi Buffer(Promega), 6 mM MgCl_2_ (Promega), 0.012 mM of each dNTP (Promega), 0.4 pM of each primer, 0.1 U/μl of GoTaq® DNA Polymerase (Promega) and 1.6 ng/μl of template DNA. Deionized water was added to complete the final volume. The thermal cycler was set at 94°C for 2 min and then 32 cycles, each of denaturation for 30 s at 94°C, annealing for 30 s at 60°C, extension for 30 s at 72°C and a final extension for 7 min at 72°C.

Biological forms of *Cx. pipiens* s.l. samples that were positive for *Dirofilaria* spp. were diagnosed with specific primers pipCQ11R (5'-CAT GTT GAG CTT CGG TGA A-3'), form *pipiens* (200 bp), and molCQ11R (5'-CCC TCC AGT AAG GTA TCA AC-3'), form *molestus* (250 bp), and universal primer CQ11F2 (5'-GAT CCT AGC AAG CGA GAA C-3') for microssatellite locus CQ11, as described by Bahnck & Fonseca, 2006 [33]. Positive controls from homozygous *Cx. pipiens* f. *pipiens* and *Cx. pipiens* f. *molestus* were used and deionized water was used as negative control.

In order to confirm that a product of 250 pb in the last mentioned PCR belongs to *Cx. pipiens* f. *molestus* and not to *Cx. quinquefasciatus*, these members of the *Culex pipiens* complex were differentiated according to polymorphisms in the intron-2 of the acetylcholinesterase-2 (*ace-2*) gene [34]. Specific primers to detect *Cx. pipiens* s.s*.* (610 pb) and *Cx. quinquefasciatus* (274 pb) were used, namely, ACEpip (5’-GGA AAC AAC GAC GTA TGT ACT-3’) and ACEquin (5’-CCT TCT TGA ATG GCT GTG GCA-3’) and universal primer B1246s (5’-TGG AGC TCC TCT TCA CGG-3’), as described by Smith & Fonseca, 2004 [34]. Genomic DNA from homozygous *Cx. pipiens* f. *pipiens* and *Cx. quinquefasciatus* and deionized water were used as positive and negative controls.

For all PCR reactions described above, amplified products were separated on 1.5% agarose gel eletrophoresis and observed under UV light.

### Sensitivity test of PCR

In order to determine the sensitivity of the PCR assay, two procedures were devised. Assays were carried out with DNA extracted from *Cx. theileri* female mosquitoes from IHMT colony, also separated into head+thorax and abdomen: **i)** to determine the minimum amount of parasite DNA that would be detected by the PCR assay, definite amounts of parasite DNA (10 ng, 5 ng, 1 ng, 0.1 ng, 10 pg and 1 pg) were mixed with 80 ng of mosquito DNA, and PCR reaction was performed in same conditions as described above. This showed that it was able to detect up to 10 pg of parasite DNA in 80 ng of mosquito DNA, either from head+thorax or abdomen; **ii)** it was also determined the sensitivity cut-off of detecting an infected mosquito in a pool of 40 mosquitoes. After the first individual specimen of *Cx. theileri* positive for *D. immitis* was detected, a sample from this pool with 80 ng/μl of total DNA, was diluted in uninfected *Cx. theileri* DNA at the same 80 ng/μl concentration. The test was started with 4 μl of the positive sample joined with 36 μl of uninfected mosquito DNA, *i.e.* 4:40, followed by 3:40, 2:40, 1:40, and 0.1:40. Parasite DNA was detected until 1:40 dilution, either for head+thorax or abdomen pools, corresponding to one single positive mosquito in a pool of 40 mosquitoes.

### DNA sequencing and analysis

Products from the first PCR described (panfilarial) were purified by QIAquick PCR Purification Kit (Qiagen) and sequenced by Macrogen. Sequences were edited and aligned using BioEdit [35], and compared to other similar sequences available in Genbank, as identified through BLAST [36].

### Calculation of the infection rate of mosquitoes

The infection rate of mosquitoes were estimated by: i) Minimum infection rate (MIR), *i.e.* the number of positive mosquito pools/total number of mosquitoes in pools tested×1000, and ii) Estimated Rate of Infection (ERI) which is adjusted for pooled samples, by the formula: ERI=1−(1- x/m)1/k where x is the number of positive pools; m the number of examined pools and k the average number of specimens in each pool [37].

### Ethical considerations

The study was approved by the Commission on Ethics of the Instituto de Higiene e Medicina Tropical, Universidade Nova de Lisboa with reference 21-2013-TM, and all procedures were performed according to national and European legislation.

### Mosquito data and statistical analysis

Mosquito densities are presented as the number of mosquitoes captured per trap-night for CDC collections, or as the number of mosquitoes collected per collector-hour for IR collections. The arithmetic mean and the standard deviation were calculated for densities per district for all collections of each type, and date. However, the median and interquartile interval (Q1-Q3) revealed to be most appropriate for this data.

Statistical analysis was carried out using the SPSS package version 20.0 for Windows [38]. Kolmogorov-Smirnov (Lilliefors modification) and Shapiro-Wilk tests were used to analyse data for normality, while Levene’s test was used to test for homogeneity of variance. Due to the lack of normality of the data, large standard deviations and lack of homogeneity of variance, non-parametric tests were used to analyse mosquito densities [39]. Mann–Whitney (MW) and Kruskal-Wallis (KW) tests were used for comparing, respectively, mosquito densities between the two years, and mosquito densities among the three districts. In the latter case, whenever significant differences were found, multiple comparisons were performed using the Dunn-Bonferroni (DB) pairwise comparisons.

Differences in mosquito rates of infection among species and locations were compared using Chi-squared test and Fisher’s exact test.

### Estimation of *Dirofilaria* development units (DDU)

In order to determine the hypothetical period in which there was risk of heartworm disease transmission in the surveyed areas, *Dirofilaria* Development Units (DDU) were calculated. For each day in which the average temperature was >14°C, temperature at which there is no extrinsic development of the parasite, the difference between the average temperature and 14°C was calculated (*i.e.* for *Tmean*≥15, DDU=*Tmean*-14) [40]. The sum of DDUs in the 30 days following the first day with average temperature >14°C, designated as DDU_30_, was then calculated. When DDU_30_ is ≥130, it is assumed that a mosquito that might have taken a blood meal on a microfilaremic host on that particular day, had the possibility of allowing the completion of the extrinsic cycle, hence becoming infective, admitting an average mosquito life span of 30 days [2,13,40], independently of temperatures lower than 14°C during that period [41]. With this data, a bar graph was plotted depicting the favourable days for the completion of the extrinsic cycle, and for the transmission of heartworm in the areas and time periods studied [42].

## Results

### Mosquito species captured and relative abundance

In total, 9156 female mosquitoes were caught in the whole sampling period (July/2011-May 2013), representing 11 species from five different genera. *Culex (Culex) theileri* was the most frequent species (5812, 63.48%), followed by *Cx. (Cux) pipiens* s.l. (1940, 21.19%), *Aedes (Ochlerotatus) caspius* (601, 6.56%), *Anopheles (Anopheles) maculipennis* s.l. (406, 4.43%), *Cx. (Cux) univittatus* (145, 1.58%), *Culiseta (Allotheobaldia) longiareolata* (30 0.33%), *Ae. (Och) detritus* s.l. (23, 0.25%), *Cs. (Culiseta) annulata* (17, 0.19%), *An. (Ano) claviger* s.l. (4, 0.04%), *Cs. (Cul) subochrea* (3, 0.03%) and *Aedes (Och) berlandi* (2, 0.02%). For 143 female mosquitoes it was not possible to identify beyond genus (*Culex* sp.) and for 30 it was not possible to distinguish between *Cx. theileri* and *Cx. univittatus*, comprising jointly 1.89% of the total collection.

The district of Santarém showed the highest number of mosquitoes captured (7818, 85.4%), followed by Coimbra (679, 7.4%) and Setúbal (659, 7.2%). Relative frequencies of the mosquito species caught in the different districts are depicted in Figure 1. *Culex theileri* was the most abundant species in Santarém and Coimbra, followed by *Cx. pipiens* s.l.. In Setúbal, the most frequent species found were *Cx. pipiens* s.l., *Cx. theileri*, *An. maculipennis* s.l. and *Ae. caspius*.

**Figure 2:**
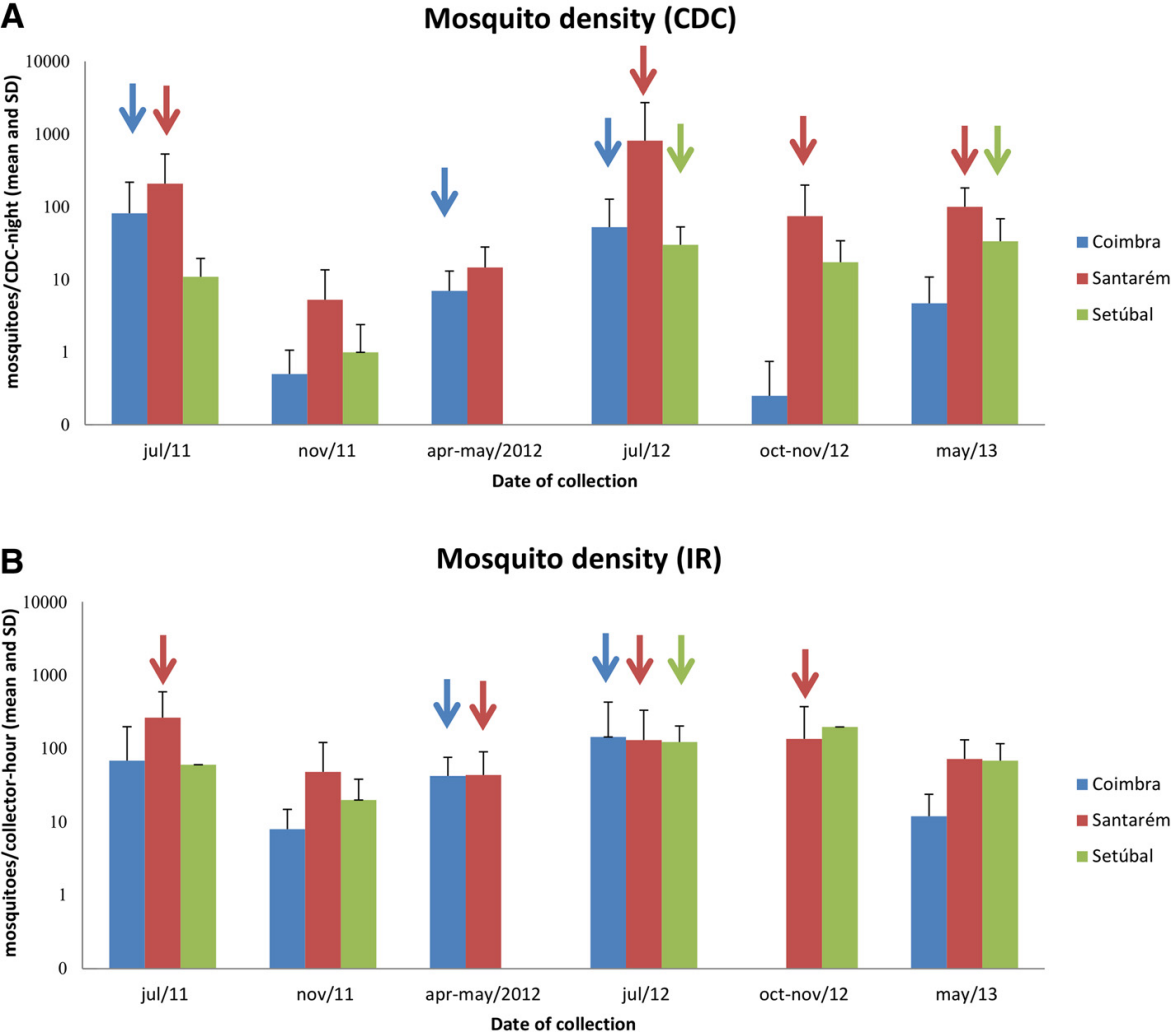
**Mosquito average density in the three sampled districts of mainland Portugal, over 2011–2013. A**- CDC trap collections; **B**- Indoor resting (IR) collections; SD - standard deviation. Arrows point to detection of infected mosquitoes.

Average mosquito densities, and respective relative frequencies, were estimated according to the collecting method (Figure 2, Table 2). For CDC traps, total mosquito densities differed among the 3 districts (KW: 14.231, DF=2, *P*=0.001) for the joint collections of the sampling period. Santarém exhibited a higher mosquito density compared just to Coimbra.

**Table 2:** **Mosquito densities in the three surveyed districts of Portugal, from July 2011 to May 2013**

**Mosquito species**	**District**					
	**Coimbra**		**Santarém**		**Setúbal**	
	**CDC**	**IR**	**CDC**	**IR**	**CDC**	**IR**
	**mean (SD); median (Q1-Q3)**	**mean (SD); median (Q1-Q3)**	**mean (SD); median (Q1-Q3)**	**mean (SD); median (Q1-Q3)**	**mean (SD); median (Q1-Q3)**	**mean (SD); median (Q1-Q3)**
*Cx. theileri*	16.3 (59.5); 0 (0–0)	10.9 (48.6); 0 (0–0)	138.4 (622.8); 1.5 (0–15.3)	7.2 (21.4); 0 (0–6)	6.2 (12.8); 1 (0–4)	4.7 (9.2); 0 (0–6.4)
*Cx. pipiens* s.l.	4.8 (7.7); 0 (0–8)	4.4 (7.5); 0 (0–6)	39.2 (70.9); 15 (5–42.5)	26.6 (54.7); 0 (0–34.7)	7.4 (8.9); 2.5 (0–12.3)	51.9 (62.8); 18 (0–84)
*Ae. caspius*	3.5 (11.5); 0 (0–2)	2.1 (5.8); 0 (0–0)	11.9 (71.3); 0 (0–0)	0.3 (1.3); 0 (0–0)	2.8 (4.4); 1 (0–2.5)	2.0 (5.5); 0 (0–0)
*An. maculipennis* s.l.	0.2 (0.5); 0 (0–0)	30.8 (117.4); 0 (0–0)	0.5 (1.5); 0 (0–0)	85.3 (187.2); 0 (0–30)	0.1 (0.2); 0 (0–0)	31.6 (69.9); 0 (0–30)
Total mosquitoes	25.2 (66.4); 3 (0–13.5)	48.8 (133.9); 6 (0–17.8)	199.2 (759.5); 22 (10.5-61)	122.5 (196.3); 42 (0–118.5)	17.4 (22.5); 12 (1.5-20.8)	90.8 (79.5); 72 (27–134.4)

Mean, standard deviation (SD) and medians and interquartile interval (Q1-Q3), of the most abundant species, as well as their total, are presented according to collecting method. CDC: CDC traps, mosquito/trap-night; IR: indoor resting, mosquito/collector-hour.

*Culex theileri* revealed different densities in the three districts (KW: 8.548, DF=2, *P*=0.014), being relatively more abundant just in Santarém compared to Coimbra.

*Culex pipiens* s.l. equally revealed different densities in the three districts (KW: 19.277, DF=2, *P*<0.001), being more abundant in Santarém compared to Coimbra, and to Setúbal.

*Aedes caspius* also revealed different densities in the three districts (KW: 12.930, DF=2, *P*=0.002), being significantly more abundant in Setúbal than in Santarém.

*Anopheles maculipennis* s.l. collected by CDC traps did not reveal differences among the three surveyed districts.

As for IR collections, these densities also differed among the 3 districts for the total of the collecting period (KW: 9.802, DF=2, *P*=0.007). Mosquito density in Setúbal was significantly higher just in relation to Coimbra.

*Culex pipiens* s.l. also differed among the 3 districts for the total of the collecting period (KW: 7.230, DF=2, *P*=0.027), being more abundant just in Setúbal compared to Coimbra.

*Anopheles maculipennis* s.l., *Cx. theileri* and *Ae. caspius* were not significantly different between the three districts, in IR collections.

As to mosquito densities on the two surveying years, CDC collections in the second year, 2012/2013: median 22.0 (12.3-50.8) mosquitoes/trap-night, were higher than in the first, 2011/2012: 5.0 (0.5-20.5) mosquitoes/trap-night (M-W: 1,094.5, *P* = 0.002). This difference was valid only for *Cx. theileri*, 2012/2013: 1.5 (0–11.5) mosquitoes/trap-night, compared to 2011/2012: 0.0 (0–1) (M-W: 1,060.0, *P*=0.003), and for *Cx. pipiens* s.l., 2012/2013: 12.5 (3–30.5) mosquitoes/trap-night, compared to 2011/2012: 1.0 (0–10.5) mosquitoes/trap-night (M-W: 1,119.0, *P*=0.001). However, indoor resting collections did not show such differences on the total mosquitoes, but only for *Cx. pipiens* s.l., 2012/2013: 8.6 (0–48) mosquitoes/collector-hour, compared to 2011/2012: 0.0 (0–0.3) mosquitoes/collector-hour (M-W: 653.0, *P*=0.005).

### Molecular detection of *D. immitis* DNA in mosquitoes

For PCR analysis, we used 5866 adult female mosquitoes. In total, 1815 head+thorax pools and 1529 abdomen pools were screened using the pan-filarial primers. This difference is due to bloodfed or semigravid females that still contained undigested blood in the abdomen, in order to avoid contamination of *Dirofilaria* spp. DNA that might be in the blood meal, thus preventing assumption of an established mosquito infection. *Dirofilaria immitis* DNA was found in the four most frequent species, but also in *Ae. detritus* s.l. (Table 3, with respective values of MIR, ERI and 95% CI).

**Table 3:** ***Dirofilaria immitis*** **DNA detected in mosquitoes collected in Portugal, 2011–2013**

**Mosquito species**	**Mosquitoes collected**	**Mosquitoes tested**		**Positive PCR**		**Number of positive pools** ^**§**^	**MIR**	**ERI**	**95% CI**
		**Specimens**	**Pools†**	**Head+thorax**	**Abdomen**				
*Cx. theileri*	5812	3406	234	13	16	23	6.8/1000	0.71%	0.005–0.01
*Cx. pipiens* s.l.	1940	1595	1123	0	8	8	5/1000	0.50%	0.003–0.01
*Ae. caspius*	601	270	193	4	8	10	37/1000	3.73%	0.02–0.067
*An. maculipennis* s.l.	406	400	114	5	8	12	30/1000	3.12%	0.017–0.052
*Ae. detritus* s.l.	23	23	16	1	1	1	43.5/1000	4.39%	0.008–0.21
Other species*	374	172	135	0	0	0	-	-	
TOTAL	9156	5866	1815	0	0	0	9.2/1000	0.91%	0.007–0.012

§ - whether in just one portion of the body or both; * - other species and unidentifiable mosquitoes; † − includes individual specimens as well.

*Culex pipiens* s.l. positive pools for *D. immitis*, were identified as *Cx. pipiens* s.s., 7 form *pipiens* and 1 form *molestus*, which was from Setúbal.

The distribution of positive mosquitoes over the three sampled districts, and their respective values of MIR, ERI and 95% CI are depicted in Table 4.

**Table 4:** ***Dirofilaria immitis*** **infection of mosquitoes by sampling districts of Portugal, 2011–2013**

**Districts**	**Mosquitoes collected**	**Mosquitoes tested**		**Positive PCR**		**Number of positive pools** ^**§**^	**MIR**	**ERI**	**95% CI**
		**Specimens**	**Pools†**	**Head+thorax**	**Abdomen**				
Coimbra	679	678	209	9	14	21	31.0/1000	3.21%	0.02–0.047
Santarém	7818	4530	1208	13	19	25	5.5/1000	0.54%	0.004–0.008
Setúbal	659	658	398	1	8	8	12.2/1000	1.22%	0.006–0.024
TOTAL	9156	5866	1815	23	41	54	9.2/1000	0.91%	0.007–0.012

§ - whether in just one portion of the body or both; † − includes individual specimens as well.

No amplicons corresponding to the diagnostic size for *D. repens* DNA were obtained.

Sequencing confirmed all filarial DNA as belonging to *D. immitis* (nucleotide sequence data are available under accession numbers [ENA: LN626262 to LN626267] [43], with a sequence similarity on BLAST [36] that ranges from 89% to 97% with sequences available at NCBI database [JX866681.1; DQO18785.1; JX866681.1; FJ263464.1; FJ2634571; HM126606.1.].

Overall, mosquitoes with *D. immitis* DNA were found in all collecting dates, but November 2011, usually by both methods and in more than one district (Figure 2, Table 5).

**Table 5:** **Occurrence of mosquitoes positive for** ***D. immitis*** **, according to species, district and date, Portugal 2011–2013**

**Mosquito species**	**2011**		**2012**			**2013**
	**JUL**	**NOV**	**APR-MAY**	**JUL**	**OCT-NOV**	**MAY**
*Cx. theileri*	CO, SA	-	-	CO, SA, SE	SA	SE
*Cx. pipiens* s.s.	CO, SA	-	-	SA	-	SA, SE
*Ae. caspius*	CO, SA	-	CO	CO	-	SE
*An. maculipennis* s.l.	SA	-	CO, SA	CO, SA, SE	SA	-
*Ae. detritus* s.l.	-	-	CO	-	-	-

CO, Coimbra; SA, Santarém; SE, Setúbal.

The overall infection rate of mosquitoes was significantly different for the three districts (*χ*^2^=40.93, Df=2, *P* <0.0001), being higher in Coimbra (3.21%), compared to Santarém (0.54%, *χ*^2^=36.11, Df=2, *P*<0.0001) or Setúbal (1.22%, *χ*^2^=19.46, Df=2, *P*<0.0001), but not significantly different between Santarém and Setúbal.

### Estimation of transmission risk of *Dirofilaria* spp. by mosquitoes

**Figure 3:**
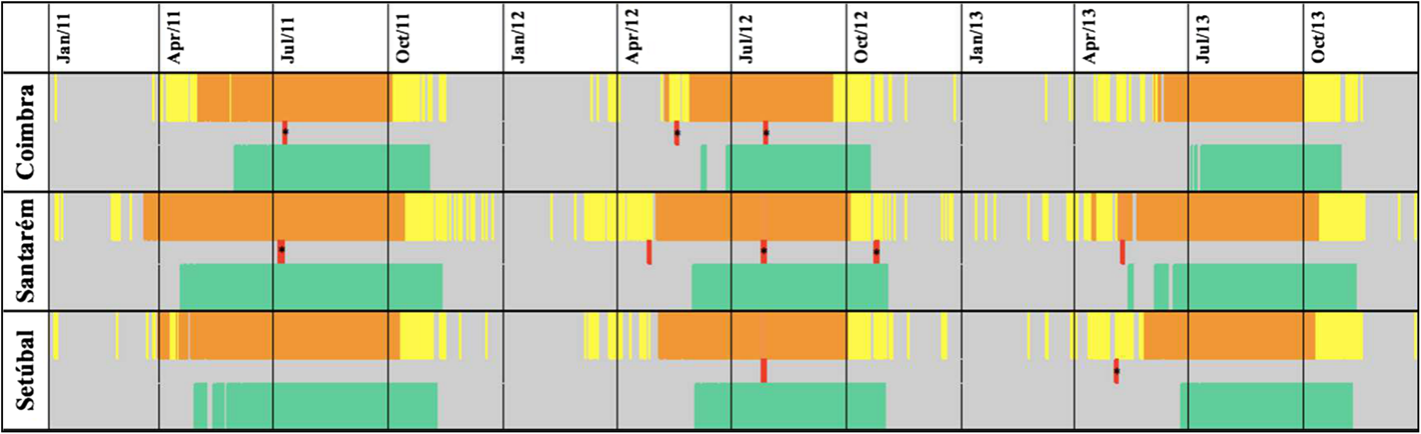
**Potential transmission period of** ***Dirofilaria*** **spp. in 2011–2013 for Coimbra, Santarém and Setúbal, Portugal.** Yellow-days with average temperature above 14°C; Orange-Days with average temperature above 14°C and at least 130 DDU_30_; Green-days with transmission risk to the vertebrate host, 30 days after the first day with 130 DDU_30_; Red bars indicate the occurrence of positive mosquito pools in this work, abdomen pools, when plain, and head+thorax pools, when marked with a black star.

The calculation of the DDU_30_ for the three studied districts showed that there was, at least, 152 days in 2011, 119 days in 2012 and 117 days in 2013 with suitable conditions for the completion of the extrinsic development of *Dirofilaria* spp., and consequently, for its transmission to the vertebrate host (Figure 3). Most of the infected mosquito pools detected in this work (red lines) are in agreement with the determined favourable development periods.

## Discussion

To our knowledge, this is the first report of molecular evidence for natural infections of mosquitoes with *D. immitis* in continental Portugal. Despite known prevalence of canine dirofilariasis (CD), the knowledge of its natural and potential vectors in mainland Portugal was scarce, with a historical study considering *Cx. theileri* as a probable vector of *Dirofilaria* spp. [25]. We report the finding of *An. maculipennis* s.l*.*, *Ae. caspius*, *Ae. detritus* s.l. and *Cx. theileri* as likely competent vectors of *D. immitis*, *i.e.* with DNA in head+thorax, and *Cx. pipiens* form *pipiens* and form *molestus*, as likely vectors, *i.e.*with DNA only in abdomens, but from mosquitoes without any traces of bloodmeal. In this work, only *D. immitis* was detected, in contrast with recent findings of *D. repens* in other Southern European countries such as Italy, albeit in much lower rates than *D. immitis*, in *Cx. pipiens* [16,17], or at similar rates as *D. immitis* in *Cx. pipiens* and *Ae. albopictus* [44]. On the other hand, this is not surprising as *D. repens* was not found in parallel animal surveys in the same districts of Portugal [24].

*Culex theileri*, *Cx. pipiens* s.l. and *An. maculipennis* s.l. have already been implicated as vectors in countries such as Spain [15,20], Italy [16,17], Turkey [18] and Iran [21]. *Aedes caspius* and *Ae. detritus* s.l. are here for the first time, to the best of our knowledge, implicated as natural competent vectors of *D. immitis. Aedes caspius* had been found positive for the whole mosquito [17], and *Ae. detritus* s.l. for the abdominal portion [45], hence requiring confirmation. This is also the first study in which both biological forms of *Cx. pipiens* s.s., form *pipiens* and form *molestus* have been found infected with *D. immitis*. In Portugal, there are to date, records of *Cx. pipiens* and *Cx. torrentium* as members of the *Cx. pipiens* complex [46]. *Culex torrentium* is rare and occurs only in northern and mountain areas of the country [46], therefore, none of the collected specimens could belong to this species. As to *Culex quinquefasciatus*, although it has not yet been recorded in Portugal, hybrids with *Cx. pipiens* have recently been found in Greece [47]. For this reason, and considering either the ongoing climatic changes and its consequences on species distribution, or the similar PCR results between form *molestus* of *Cx. pipiens* s.s. and *Cx. quinquefasciatus*, all specimens were treated as *Cx. pipiens* s.l.; the molecular identification being made only for positive specimens for *D. immitis*.

*Anopheles maculipennis* s.l. has also been previously found infected with *D. immitis* [16]. However, *An. atroparvus* is the only member of this complex occurring south of the Montejunto-Estrela mountain range, and even to the north of this range a proportion of nine *An. atroparvus* to one *An. maculipennis* s.s. was found [46,48,49]. Thus, we can be confident that the positive *An. maculipennis* s.l. in the district of Setúbal are in fact *An. atroparvus,* hence becoming the first vector incrimination for this species.

*Aedes detritus* s.l. were not differentiated as the technique available at the time of this study would preclude the screening for dirofilarial DNA.

Infection rates were similar whether estimated as MIR or ERI, probably as a great proportion of our pools were of a single mosquito specimen. The species with the highest infection rates were *Ae. caspius* (3.7%), followed by *An. maculipennis* s.l. (3.1%), *Cx. theileri* (0.7%) and *Cx. pipiens* s.l. (0.5%). The highest infection rate was in fact recorded for *Ae. detritus* s.l.. However, infection rates based on small sample sizes, *i.e.* <1000, may not accurately represent the true infection rate in the population [50]. Whereas in the case of *An. maculipennis* s.l. and *Ae. caspius* the sample size is 400 and 270, respectively, with somewhat large 95% CIs, and therefore, infection rates should be interpreted with caution, in the case of *Ae. detritus* s.l., with a sample of 23 specimens and a much wider 95% CI, the very high infection rate has a reduced significance.

Infection of *Cx. pipiens* in Spain has been detected with a rate of 0.3% [15], in Italy it ranges from 0.048% [17], much lower than this present study, to 0.26% and 0.54% [16,44], similar to our values, with an intermediate prevalence rate in Turkey of 0.12% [18]. Infection rates in *Ae. caspius* in Italy were much lower than ours, 0.18% [17]. Still in Italy, 5.26% (1/19) of *An. maculipennis* s.l. were found infected. However, much higher infection rates for *An. maculipennis*, 11.7% (4/34) and *Cx. theileri*, 10% (15/149) were found in Iran [21], albeit several of these values were based on small sample sizes.

In Portugal, previous detection of *Dirofilaria* spp. L1 and L2 larvae in the MT of *Cx. theileri*, in the district of Setúbal, had yielded an infection rate of 4.76%, again, in a small sample (N=42) [25]. In islands of Macaronesia, *Cx. theileri* has been found at infection rates of 0.16% in the Canaries [20] and *circa* 0.9%-1.13% in Madeira [19], to which the values in this study are more approximate.

In continental Portugal there are 41 identified species of mosquitoes [46], however, *An. maculipennis* s.l*.*, *Cx. pipiens* s.l., *Cx. theileri* and *Ae. caspius* are the most abundant and broadly distributed [26]. In the three districts surveyed in this work, these were also the species with highest densities. Total mosquito densities were lower in Coimbra, the northern most district, in agreement with previous surveys, namely for *Cx. pipiens* s.l., by both methods, as a sign of identical capture yield for this species [26]. Conversely, CDC trap catches were able to show different densities among districts, for *Cx. theileri* and *Ae. caspius*, as they are superior for targeting these species [26]. On the other hand, IR catches yielded higher numbers of *An. maculipennis* s.l., as IR tends to be a more adequate method to capture this species [51]. Nevertheless, in this study, a striking difference was reported in the mosquito abundance in the district of Setúbal, with much lower densities compared to previous works [26,52,53]. The reasons contributing to this may well be i) the location of the collecting sites close to kennels, as per the experimental design, and which in this district were in areas not favourable for mosquito breeding, as opposed to earlier works which included rice fields and wetlands; ii) relatively low number of collections, with only one set in peak breeding season; and iii) strong winds registered in some of the collecting dates, as noted in field collection registers.

Collections in the second year yielded higher densities, particularly for *Cx. theileri* and *Cx. pipiens* s.l.. This increase may be due to local environmental variables, particularly climatic. Although no significant differences were registered for the average temperatures, precipitation was higher in the three districts in the second sampling year, 2012/2013. Nevertheless, considering there were only three collecting moments per year, there is not enough data to draw conclusions on the seasonal dynamics of mosquitoes.

Infection rates in mosquitoes were not in agreement with prevalence rates of CD found in the same research project [24], despite having targeted mosquito collections to the vicinity of kennels. Highest and lowest infection rates for mosquitoes were registered in Coimbra and Santarém, respectively, which had similar prevalence rates of CD. On the other hand, Setúbal, which had the highest CD prevalence rate, registered an intermediate mosquito infection rate. There are many factors whose influence is still unknown (vector efficiency of each species, overall level of protection in the dog population by preventive therapy, local environmental conditions in studied areas, etc.).

During the two year survey, infected mosquitoes were found in five of the six collection dates, representing an almost continuous presence of infected vectors, particularly in Santarém. Furthermore, infected mosquitoes were found by both methods in most of the collecting dates and sites. It can be argued that infected mosquitoes in IR collections may have become infected in the hosts in the shelters, however, the CDC trap collected mosquitoes represent the mosquito population searching for hosts and are proof therefore of circulating infected vectors. Coimbra was the only district with the five infected species as *Ae. detritus* s.l. was only found infected there, while Santarém and Setúbal registered four infected species.

The calculation of the DDU_30_ ranged from 117 days in 2013 to 152 days in 2011 with favourable temperatures for the completion of the extrinsic cycle of *D. immitis*, hence the existence of infective mosquito vectors that would complete the transmission cycle. Most of the infective and infected mosquitoes detected were collected during these favourable periods. The few exceptions are probably due to their maintenance in the insectary for the seven day period, which proves highly important in such studies. Although a 7 day period at 25°C may not be enough for the completion of the extrinsic cycle, a compromise had to be taken to compensate for mosquito mortality and filarial DNA degradation, while allowing for complete digestion of bloodmeals.

Activity of these mosquitoes, whether infected, infective or neither, was found in the three time point collections, corresponding to Spring, Summer and Autumn. In the context of climate changes, particularly in Portugal, where temperature increases have reached 0.5°C/decade since 1970, more than twice higher than the global median temperature [54], and with future scenarios that may range between 3–5.8°C by 2040–2090, the activity period for mosquitoes, and hence mosquito-borne diseases are likely to increase [55]. This is further relevant as dirofilariasis is recognized as an expanding zoonosis, particularly in Europe [4,5]. Our results are in agreement with predictions of occurrence and seasonality of *Dirofilaria* spp., with peaks of infection in Summer, from June to September, even in countries of Northern Europe [2,13,14,42].

## Conclusions

We have confirmed and reported new mosquito vectors of dirofilariasis in three districts of Portugal with high prevalence of CD. To our knowledge, the present study is the first PCR screening for *Dirofilaria* spp. in mosquitoes for continental Portugal. Our results confirm that not only *Cx. theileri* is capable of becoming infected with *D. immitis,* but also *Ae. caspius*, *An. maculipennis* s.l., *An. atroparvus*, *Cx. pipiens* of both bioforms *pipiens* and *molestus* and *Ae. detritus* s.l. can support the development of *D. immitis,* and with the exception of *Cx. pipiens,* to the L3 infective stage, based on the presence of filarial DNA in the head+thorax. Most of these results were in agreement with the prediction of 130 DDU_30_ for the regions surveyed. The finding of infected and infective mosquitoes in the three districts and in the Spring-Autumn interval heightens the necessity for prophylactic protective measures to prevent transmission at least during this period. Further studies are necessary to ascertain whether transmission season is wider than the interval Spring-Autumn.

## References

[1] Anderson RC (2000). Nematode parasites of vertebrates. Their development and transmission.

[2] Genchi C, Rinaldi L, Cascone C, Mortarino M, Cringoli G (2005). Is heartworm really spreading in Europe?. Vet Parasitol.

[3] McCall JW, Genchi C, Kramer LH, Guerrero J, Venco L (2008). Heartworm disease in animals and humans. AdvParasitol.

[4] Traversa D, Cesare A, Conboy G (2010). Canine and feline cardiopulmonary parasitic nematodes in Europe: emerging and underestimated. Parasites Vectors.

[5] Simón F, Siles-Lucas M, Morchón R, González-Miguel J, Mellado I, Carretón E (2012). Human and animal dirofilariasis: the emergence of a zoonotic mosaic. ClinMicrobiol Rev.

[6] Miterpáková M, Antolová D, Hurníková Z, Dubinský P, Pavlacka A, Németh J (2010). *Dirofilaria* infections in working dogs in Slovakia. J Helminthol.

[7] Jacso O, Mandoki M, Majoros G, Petsch M, Mortarino M, Genchi C (2009). First autochthonous *Dirofilaria immitis* (Leidy, 1856) infection in a dog in Hungary. Helminthologia.

[8] Świątalska A, Demiaszkiewicz AW (2012). First autochthonous case of *Dirofilaria immitis* invasion in dog in Poland. ŽycieWeterynaryjne.

[9] Szénási Z, Kovács AH, Pampiglione S, Fioravanti ML, Kucsera I, Tánczos B (2008). Human dirofilariosis in Hungary:an emerging zoonosis in central Europe. Wien KlinWochenschr.

[10] Cielecka D, Zarnowska-Prymek H, Masny A, Salamatin R, Wesolowska M, Golab E (2012). Human dirofilariosis in Poland: the first cases of autochthonous infections with Dirofilaria repens. Ann AgricEnviron Med.

[11] Sałamatin RV, Pavlikovska TM, Sagach OS, Nikolayenko SM, Kornyushin VV, Kharchenko VO (2013). Human dirofilariasis due to *Dirofilaria repens* in Ukraine, an emergent zoonosis: epidemiological report of 1465 cases. Acta Parasitol.

[12] Tasić-Otašević SA, Gabrielli SV, Tasić AV, Miladinovićtasić NL, Kostić JT, Ignjatović AM (2014). Seroreactivity to *Dirofilaria* antigens in people from different areas of Serbia. BMC Infect Dis.

[13] Genchi C, Rinaldi L, Mortarino M, Genchi M, Cringoli G (2009). Climate and *Dirofilaria* infection in Europe. Vet Parasitol.

[14] Medlock LM, Barras I, Kerrod E, Taylor MA, Leach S (2007). Analysis of climatic predictions for extrinsic incubation of *Dirofilaria* in the United Kingdom. Vector Borne Zoon Dis.

[15] Morchón R, Bargues MD, Latorre JM, Melero-Alcíbar R, Pou-Barreto C, Mas-Coma S (2007). Haplotype H1 of *Culex pipiens* implicated as a natural vector of *Dirofilaria immitis* in an endemic area of western Spain. Vector Borne Zoon Dis.

[16] Cancrini G, Magi M, Gabrielli S, Arispici M, Tolari F, Dell’Omodarme M (2006). Natural vectors of dirofilariasis in rural and urban areas of the Tuscan region, central Italy. J Med Entomol.

[17] Latrofa MS, Montarsi F, Ciocchetta S, Annoscia G, Dantas-Torres F, Ravagnan S (2012). Molecular xenomonitoring of *Dirofilaria immitis* and *Dirofilaria repens* in mosquitoes from north-eastern Italy by real-time PCR coupled with melting curve analysis. Parasites Vectors.

[18] Yildirim A, Inci A, Duzlu O, Biskin Z, Ica A, Sahin I (2011). *Aedesvexans* and *Culex pipiens* as potential vectors of *Dirofilaria immitis* in Central Turkey. Vet Parasitol.

[19] Santa-Ana M, Khadem M, Capela R (2006). Natural infection of *Culex theileri* (Diptera, Culicidae) with *Dirofilaria immitis* (Nematoda, Filarioidea) on Madeira Island, Portugal. J Med Entomol.

[20] Morchón R, Bargues MD, Latorre-Estivalis JM, Pou-Barreto C, Melero-Alcibar R, Moreno M (2011). Molecular Characterization of Culex theileri from Canary Islands, Spain, a potential vector of Dirofilaria immitis. J Clinic Experiment Pathol.

[21] Azari-Hamidian S, Yaghoobi-Ershadi MR, Javadian E, Abai MR, Mobedi I, Linton YM (2009). Distribution and ecology of mosquitoes in a focus of in northwestern Iran, with the first finding of filarial larvae in naturally infected local mosquitoes. Med Vet Entomol.

[22] Cancrini G, Ricci I, Tessarin C, Gabrielli S, Pietrobelli M (2003). *Aedesalbopictus* is a natural vector of *Dirofilaria immitis* in Italy. Vet Parasitol.

[23] Vieira L, Silvestre-Ferreira AC, Fontes-Sousa AP, Balreira AC, Morchón R, Carretón E (2014). Seroprevalence of heartworm (*Dirofilaria immitis*) in feline and canine hosts from central and northern Portugal. JournHelminth.

[24] Alho AM, Landum M, Ferreira C, Meireles J, Gonçalves L, Madeira de Carvalho L etal. Prevalence and seasonal variations of canine dirofilariosis in Portugal. Vet Parasitol 2014, doi:10.1016/j.vetpar.2014.08.014. 10.1016/j.vetpar.2014.08.014 25440945

[25] Ribeiro H, Ramos HC, Pires CA (1983). Contribuição para o estudo dos vectores de filaríases animais em Portugal. J Socied Ciências Médicas.

[26] Almeida APG, Galão RP, Sousa CA, Novo MT, Parreira R, Pinto J (2008). Potential mosquito vectors of arboviruses in Portugal: species, distribution, abundance and West Nile infection. Transact Royal Soc Trop Med Hygiene.

[27] Instituto Português do Mar e da Atmosfera https://www.ipma.pt/.

[28] Ribeiro H, Ramos HC (1999). Identification keys of the mosquitoes of continental Portugal, Azores and Madeira. Eur Mosquito Bull.

[29] Favia G, Lanfrancotti A, Della Torre A, Cancrini G, Coluzzi M (1996). Polymerase chain reaction identification of *Dirofilaria repens* and *Dirofilaria immitis*. Parasitology.

[30] Gomes B, Sousa C, Novo M, Freitas FB, Alves R, Côrte-Real AR (2009). Asymmetric introgression between sympatric molestus and pipiens forms of *Culex pipiens* (Diptera: Culicidae) in the Comporta region. Portugal BMC EvoBiol.

[31] Stothard JR, Hughes S, Rollinson D (1996). Variation within the Internal Transcribed Spacer (ITS) of ribosomal DNA genes of intermediate snail hosts within the genus *Bulinus* (Gastropoda: Planorbidae). Acta Trop.

[32] Rishniw M, Barr SC, Simpson KW, Frongillo MF, Franz M, Dominguez Alpizar JL (2006). Discrimination between six species of canine microfilariae by a single polymerase chain reaction. Vet Parasitol.

[33] Bahnck CM, Fonseca DM (2006). Rapid assay to identify the two genetic forms of *Culex (Culex) pipiens* L. (Diptera: Culicidae) and hybrid populations. Amer J Trop Med Hygiene.

[34] Smith JL, Fonseca DM (2004). Rapid assays for identification of members of the *Culex (Culex) pipiens* complex, their hybrids, and other sibling species (Diptera: culicidae). Amer J Trop Med Hygiene.

[35] Hall TA (1999). BioEdit: a user-friendly biological sequence alignment editor and analysis program for Windows 95/98/NT. Nucl Acids SympSer.

[36] BLAST® Basic Local Alignment Search Tool. http://blast.ncbi.nlm.nih.gov/Blast.cgi.

[37] Cowling DW, Gardner IA, Johnson WO (1999). Comparison of methods for estimation of individual-level prevalence based on pooled samples. Prev Vet Med.

[38] SPSS for Windows ® Inc., Statistical Package for Social Sciences®, IBM, Chicago, Illinois, USA. 1999

[39] Siegel S, Castellan NJ (1988). Nonparametric Statistics for the Behavioral Sciences.

[40] Fortin JF, Slocombe JOD (1981). Temperature requirements for the development of *Dirofilaria immitis* in *Aedes triseriatus* and *Ae. vexans*. Mosq News.

[41] Venco L, Genchi M, Genchi C, Gatti D, Kramer L: Can heartworm prevalence in dogs be used as provisional data for assessing the prevalence of the infection in cats? Vet Parasitol. 2011, 22;176 :300–3. doi: 10.1016/j.vetpar.2011.01.013. 10.1016/j.vetpar.2011.01.013 21292401

[42] Sassnau R, Czajka C, Kronefeld M, Werner D, Genchi C, Tannich E, Kampen H: Dirofilaria repens and Dirofilaria immitis DNA findings in mosquitoes in Germany: temperature data allow autochthonous extrinsic development. Parasitol Res 2014, doi 10.1007/s00436-014-3970-1. 10.1007/s00436-014-3970-1 24906992

[43] ENA ‘European Nucleotide Archive’ http://www.ebi.ac.uk/ena/data/view/LN626257-LN626267. accessed March/April 2015.

[44] Cancrini G, Scaramozzino P, Gabrielli S, di Paolo M, Toma L, Romi R (2007). *Aedes albopictus* and *Culex pipiens* implicated as natural vectors of *Dirofilaria repens* in central Italy. J Med Entomol.

[45] Cancrini G, Gabrielli S, Genchi C, Rinaldi L, Cringoli G (2007). Vectors of *Dirofilaria* nematodes: biology, behaviour and host/parasite relationships. Dirofilariaimmitis and D. repens in dog and cat and human infection.

[46] Ribeiro H, Ramos HC, Pires CA, Capela RA. An annotated checklist of the mosquitoes of continental Portugal (Diptera, Culicidae) . Actas do III Congresso Ibérico de Entomologia [Proceedings of the III Iberian Congress of Entomology], 1988, 233–254.

[47] Shaikevich E, Vinogradova E (2013). The discovery of a hybrid population of mosquitoes of the Culex pipiens L. complex (Diptera, Culicidae) on the Kos Island (Greece) by means of molecular markers. Entomologicheskoe Obozrenie.

[48] Sousa CA (2008). Malaria vectorial capacity and competence of Anopheles atroparvus Van Thiel, 1927 (Diptera: Culicidae): Implications for the potential re-emergence of malaria in Portugal. PhD Thesis.

[49] Capinha C, Gomes E, Reis E, Rocha J, Sousa CA, Do Rosário VE (2009). Present habitat suitability for *Anopheles atroparvus* (Diptera, Culicidae) and its coincidence with former malaria areas in Mainland Portugal. Geospatial health.

[50] Walter SD, Hildreth SW, Beaty BJ (1980). Estimation of infection rates in population of organisms using pools of variable size. Am J Epidemiol.

[51] Lourenço PM, Sousa CA, Seixas J, Lopes P, Novo MT, Almeida AP (2011). *Anopheles atroparvus* density modeling using MODIS NDVI in a former malarious area in Portugal. J Vector Ecol.

[52] Almeida APG, Freitas FB, Novo MT, Sousa CA, Rodrigues JC, Alves R (2010). Mosquito surveys and West Nile virus screening in two different areas of Southern Portugal, 2004–2007. Vector Borne Zoon Dis.

[53] Osório HC, Amaro F, Zé-Zé L, Moita S, Labuda M, Alves MJ (2008). Species composition and dynamics of adult mosquitoes of Southern Portugal. Eur Mosquito Bull.

[54] Ramos AM, Trigo RM, Santo FE (2011). Evolution of extreme temperatures over Portugal: recent changes and future scenarios. Clim Res.

[55] Casimiro E, Calheiros J, Santos FD, Kovats S (2006). National assessment of human health effects of climate change in Portugal: approach and key findings. Environ Health Perspect.

